# Celiac Artery Compression Syndrome in a Middle-age Woman Treated Laparoscopically

**DOI:** 10.7759/cureus.5582

**Published:** 2019-09-06

**Authors:** Gabriel O Ologun, Hannah Snyder, Cara Hannigan, Daniel Njoku, Mustafa Aman

**Affiliations:** 1 General Surgery, Robert Packer Hospital/Guthrie Clinic, Sayre, USA; 2 Medicine, Guthrie Clinic/Robert Packer Hospital, Sayre, USA; 3 Biological Science, Howard University, Washington, DC, USA; 4 Surgery, Guthrie Robert Packer Hospital, Sayre, USA

**Keywords:** arterial occlusive disease, median arcuate ligament, abdominal pain, celiac artery, celiac artery compression syndrome

## Abstract

Celiac artery compression syndrome is an anatomic compression of the celiac axis by the median arcuate ligament. Patients often present with postprandial pain, as well as symptoms of gastroparesis. It is usually a diagnosis of exclusion. The treatment is aimed at relieving the compression of the celiac artery, and neurolysis. We present a case of a 55-year-old woman with celiac artery compression syndrome treated by laparoscopic decompression of the median arcuate ligament.

## Introduction

Celiac artery compression syndrome is an anatomical compression of the celiac axis by the median arcuate ligament and diaphragmatic crura. This compression results in a constellation of symptoms such as nausea, vomiting, weight loss, and postprandial epigastric pain [1]. Celiac artery compression was first described anatomically in 1917 by Lipshutz. It was observed, in cadaveric dissections, that the celiac artery was sometimes overlapped by the diaphragmatic crura [2]. Patients with this syndrome, usually present with symptoms of non-specific abdominal pain, gastroparesis, and weight loss. It is a diagnosis of exclusion. Herein, we present a case of a 55-year-old woman with celiac artery compression syndrome treated by laparoscopic decompression of the median arcuate ligament. Informed consent was obtained.

## Case presentation

**Figure 1:**
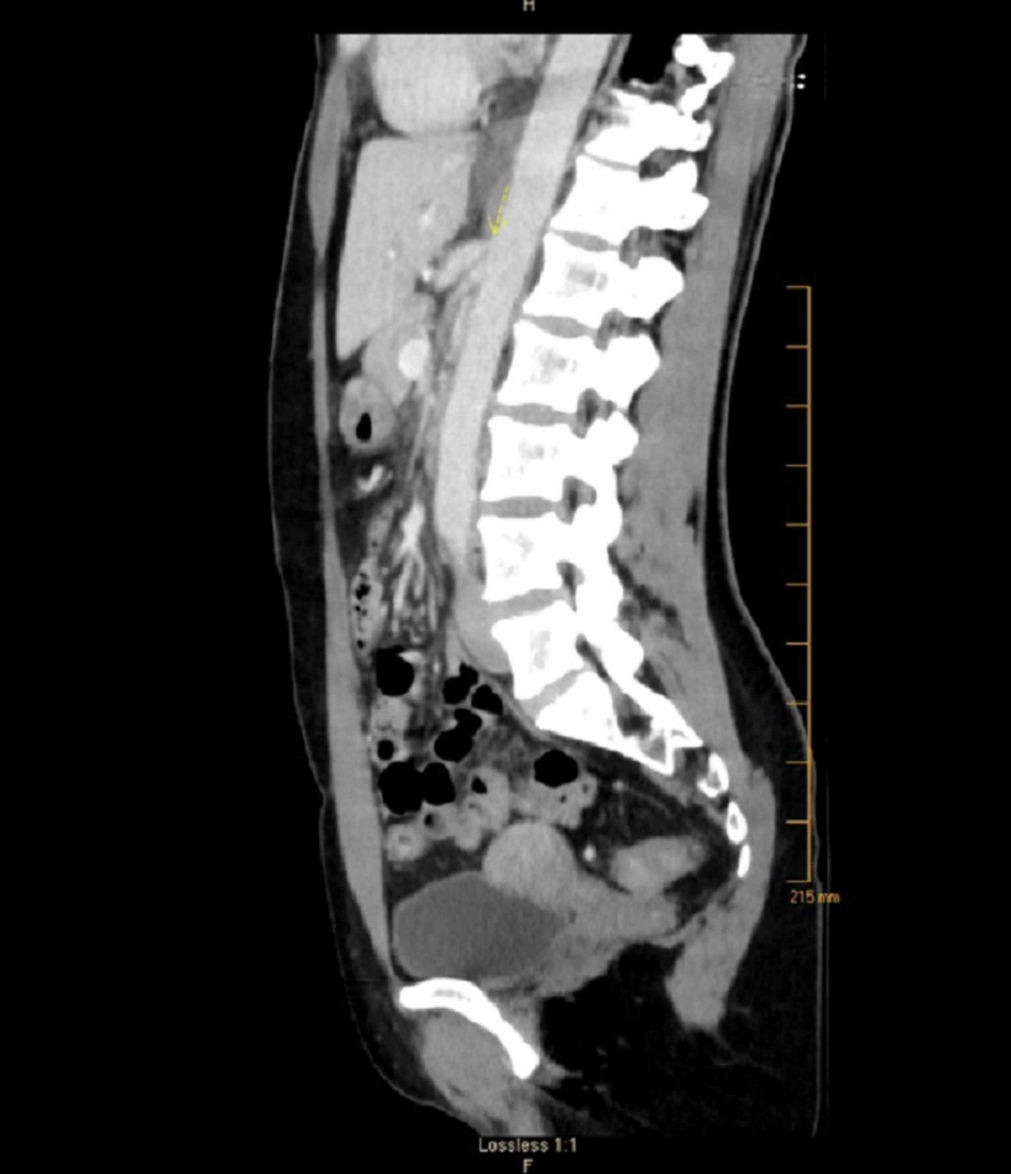
Computed tomography scan, sagittal view, showing severe stenosis of the celiac artery at its take-off from the abdominal aorta (arrow)

A 55-year-old woman was referred to our general surgery clinic for evaluation of chronic epigastric postprandial pain. Her symptoms also included nausea, vomiting, and diarrhea, with intermittent episodes of bloody bowel movements. These symptoms have been going on for several years. She had about 84 pounds (lbs) weight loss due to her inability to tolerate oral intake. She was occasionally using analgesic medications that only temporarily alleviated her abdominal pain. Personal history revealed that she had Raynaud's disease and chronic fatigue syndrome. Her home medications included baby aspirin, multivitamin, and over-the-counter Tylenol. Her physical examination along with laboratory blood tests and stool studies were unremarkable. Abdominal ultrasonography, esophagogastroduodenoscopy, and colonoscopy were unremarkable. However, computed tomography (CT) scan of the abdomen and pelvis revealed severe compression of the proximal celiac axis, near its origin, with stenosis, and post-stenotic dilatation, concerning for median arcuate ligament compression (Figure 1).

**Figure 2:**
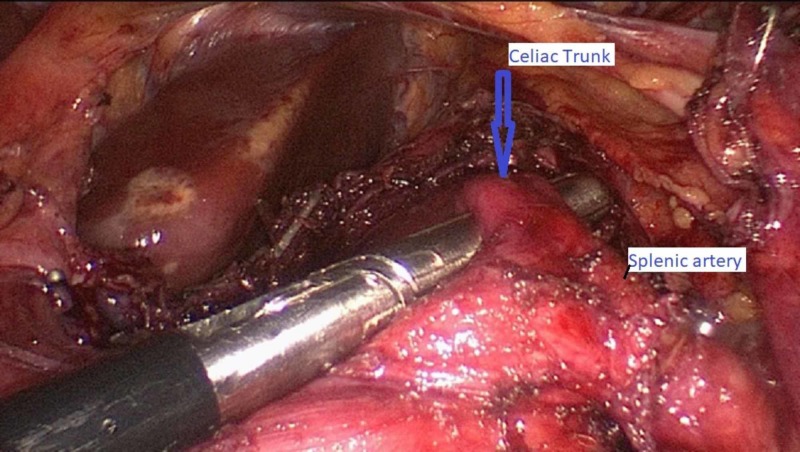
Intraoperative transection of the median arcuate ligament, arrow showing decompressed celiac trunk

After adequate preoperative assessment, laparoscopic adhesiolysis with transection of the median arcuate ligament was performed (Figure 2). Postoperatively, the patient was without complications, her postprandial abdominal pain was resolved, and she tolerated oral intake without nausea or vomiting. She was discharged home on postoperative day two. Eight weeks after discharge, at the time of this report, the patient continued to do well, tolerated regular diet without postprandial pain, and she is having formed bowel movements.

## Discussion

Several theories have been proposed on the pathophysiologic mechanism of celiac artery compression syndrome. A commonly accepted theory suggests that increased demand for blood flow, through a compressed celiac artery, leads to foregut ischemia and results in the development of epigastric pain [1]. However, the development of collateral vessels helps prevent the development of ischemia. A second hypothesis is that the pain associated with median arcuate ligament syndrome has a neuropathic component, resulting from a combination of chronic compression, and overstimulation of the celiac ganglion. This, in turn, results in the direct irritation of sympathetic pain fibers and/or splanchnic vasoconstriction and ischemia [1]. The pathophysiologic mechanism of celiac artery compression syndrome is, however, confounded by the high prevalence of asymptomatic patients exhibiting radiographic evidence of celiac artery compression [1,3]. Affected patients often present with complaints of postprandial pain, often in the upper abdomen. Some patients present with symptoms of gastroparesis: nausea, vomiting, weight loss, and epigastric bruit [4]. The symptoms of celiac artery compression syndrome closely mimic those of other abdominal disorders. The diagnosis is made by excluding other likely causes of postprandial epigastric abdominal pain, such as gastritis, gastric ulcer disease, symptomatic gallstones, or pancreatitis. Imaging modalities that can assist in the diagnosis include computed tomographic angiography, Doppler ultrasound, with the possible addition of other studies such as mesenteric duplex ultrasonography, magnetic resonance angiography, gastric tonometry, and mesenteric arteriography [5]. The management of celiac artery compression syndrome is aimed at relieving the compression of the celiac artery to restore adequate blood flow through the vessel and neurolysis to address the chronic abdominal pain [1]. Open surgical decompression is one approach to treating celiac artery compression syndrome. It involves an open midline laparotomy to access and decompress the median arcuate ligament and the diaphragmatic crura away from the celiac artery. The diaphragmatic fibers are incised to expose the aorta. The diagnosis can be confirmed intraoperatively, by visual inspection, and duplex ultrasonography demonstrating a return to normal peak systolic velocities. Neurolysis and wide excision of the involved celiac plexus are also recommended for addressing the neuropathic component of the chronic compression [1]. A second approach, to treating celiac artery compression syndrome, is laparoscopic decompression. The benefits of laparoscopic decompression include small incisions, decrease postoperative morbidity (such as ileus, pain, blood loss, adhesions, and shorter recovery time), and improved view of the surgical field. The disadvantage includes difficulty in controlling hemorrhage, potential for incomplete decompression, and increased risk of injury to the abdominal aorta due to difficult laparoscopic dissection [1,4]. A third approach, in the surgical management of celiac artery compression syndrome, is the robotic-assisted technique. The benefits of this technique include optic enhancement (increased magnification of structures, three-dimensional view), and operator-based improvement (tremor elimination, added degree of motion, and scaled operator movements). The limitations include longer operating time, additional training for the surgeon, and increased cost [1,5]. Of note, endovascular intervention-arteriography and percutaneous transluminal angioplasty, although not a successful first-line therapy in the treatment of celiac artery compression syndrome, with the addition of a balloon-expandable stent does serve as an adjunct in the management of patients with residual symptoms, and/or stenosis after an operative intervention [1].

## Conclusions

Celiac artery compression syndrome is an uncommon cause of postprandial abdominal pain. It is a diagnosis of exclusion. Management is aimed at relieving the compression of the celiac artery and neurolysis, and this can be achieved with either an open, laparoscopic, or robotic-assisted technique.
